# Genetics of Tinnitus: An Emerging Area for Molecular Diagnosis and Drug Development

**DOI:** 10.3389/fnins.2016.00377

**Published:** 2016-08-19

**Authors:** Jose A. Lopez-Escamez, Thanos Bibas, Rilana F. F. Cima, Paul Van de Heyning, Marlies Knipper, Birgit Mazurek, Agnieszka J. Szczepek, Christopher R. Cederroth

**Affiliations:** ^1^Otology and Neurotology Group, Department of Genomic Medicine, Pfizer - Universidad de Granada - Junta de Andalucía Centro de Genómica e Investigación Oncológica, PTSGranada, Spain; ^2^Department of Otolaryngology, Instituto de Investigación Biosanitaria ibs.GRANADA, Complejo Hospital Universitario GranadaGranada, Spain; ^3^1st Department of Otolaryngology, National and Kapodistrian University of Athens, Hippocrateion HospitalAthens, Greece; ^4^Ear Institute, UCLLondon, UK; ^5^Department of Clinical Psychological Science, Maastricht UniversityMaastricht, Netherlands; ^6^University Department ENT and Head and Neck Surgery, Antwerp University Hospital, University of AntwerpAntwerp, Belgium; ^7^Hearing Research Centre Tübingen, Molecular Physiology of HearingTübingen, Germany; ^8^Tinnitus Center, Charité-Universitätsmedizin BerlinBerlin, Germany; ^9^Department of ORL, Charité-Universitätsmedizin BerlinBerlin, Germany; ^10^Experimental Audiology, Department of Physiology and Pharmacology, Karolinska InstitutetStockholm, Sweden

**Keywords:** epidemiology, genetic, hearing loss, tinnitus, meniere's disease, phenotyping, subtype

## Abstract

Subjective tinnitus is the perception of sound in the absence of external or bodily-generated sounds. Chronic tinnitus is a highly prevalent condition affecting over 70 million people in Europe. A wide variety of comorbidities, including hearing loss, psychiatric disorders, neurodegenerative disorders, and temporomandibular joint (TMJ) dysfunction, have been suggested to contribute to the onset or progression of tinnitus; however, the precise molecular mechanisms of tinnitus are not well understood and the contribution of genetic and epigenetic factors remains unknown. Human genetic studies could enable the identification of novel molecular therapeutic targets, possibly leading to the development of novel pharmaceutical therapeutics. In this article, we briefly discuss the available evidence for a role of genetics in tinnitus and consider potential hurdles in designing genetic studies for tinnitus. Since multiple diseases have tinnitus as a symptom and the supporting genetic evidence is sparse, we propose various strategies to investigate the genetic underpinnings of tinnitus, first by showing evidence of heritability using concordance studies in twins, and second by improving patient selection according to phenotype and/or etiology in order to control potential biases and optimize genetic data output. The increased knowledge resulting from this endeavor could ultimately improve the drug development process and lead to the preventive or curative treatment of tinnitus.

## Introduction

Tinnitus, the perception of a phantom sound, affects nearly 15% of the population. It can severely affect quality of life in 3–6% of the population, becoming chronically bothersome, and incapacitating (Davis and Refaie, 2000). From the social perspective, tinnitus leads to a loss of productivity and increases the risk of receiving a disability pension (Friberg et al., 2012). Tinnitus varies on the perceptional level, ranging from beeping, hissing, ringing, and buzzing to drumming sounds. Tinnitus can be objective (generated by the ear and perceived by external people) or subjective (only perceived by the concerned individual), pulsatile (synchronous or asynchronous), constant or intermittent, loud or faint, perceived in one or both ears, or within the head. Despite the fact that noise overexposure is most frequently associated with tinnitus (15%) (Nicolas-Puel et al., 2006), tinnitus may be associated with many conditions other than dysfunction of the auditory system (e.g., obesity, diabetes, smoking, alcohol consumption, neck pain, allergies, thyroid dysfunction, brain tumors, temporomandibular joint (TMJ) dysfunction and as a side effect of several medications) (Baguley et al., 2013). Tinnitus often coincides with severe psychological dysfunction. Anxiety, depression, and disruptions in the execution of cognitive and attention tasks are frequently reported. Another symptom commonly associated with tinnitus is a decreased tolerance to loud sounds (hyperacusis), which is observed in 40–55% of patients with tinnitus (Baguley, 2003; Schecklmann et al., 2014). Tinnitus can be categorized based on psychoacoustic features and the levels of severity, psychological distress, and daily life disability. According to its duration, tinnitus is often assessed as follows: up to 3 months of duration is considered “acute” between 3 and 12 months “subacute” and more than 1 year is considered “chronic.” At present, there are no effective drugs for tinnitus while the need for effective treatments is likely to increase (Cederroth et al., 2013). The lack of treatment success in clinical trials has been attributed to the heterogeneity of clinical conditions associated with tinnitus. Genetic studies would help in identifying diagnostic markers for subgroups of tinnitus patients (subtypes) or markers of resistance to treatment in order to improve the selection of subjects and optimize treatment outcome. In addition, since the current pipeline of drugs to treat tinnitus is rather small (Cederroth et al., 2013), genetic studies could provide additional targets for drug development.

In this article, we briefly present the current evidence regarding heritability in tinnitus, the hypothetical pathophysiological mechanisms of tinnitus and the underlying challenges of tinnitus phenotyping. We next propose different approaches toward the genetic elucidation of tinnitus including the analysis of concordance in twins, familial aggregation studies, exome sequencing in families with multiple cases, and sequencing studies in cohorts of patients with extreme phenotypes. We suggest that tinnitus subtyping strategies based on precise definition of phenotypes would favor the selection of homogeneous groups of tinnitus patients with matching controls that might serve as a solid basis for genetic studies.

## Genetic contribution to tinnitus: the missing evidence

There is a lot of evidence to support a genetic contribution for complex disorders: differences in the prevalence according to the ethnic background, familial aggregation, and higher concordance in monozygotic twins than in dizygotic twins. In this section, we address each of these in the context of tinnitus.

The prevalence of tinnitus ranges from 6 to 30%, while the prevalence of severe tinnitus ranges from 0.7 to 16% in the same studies (Cooper, 1994; Sindhusake et al., 2003; Hasson et al., 2010; Krog et al., 2010; Michikawa et al., 2010; Nondahl et al., 2010; Shargorodsky et al., 2010; Engdahl et al., 2012; McCormack et al., 2014; Park et al., 2014; Gallus et al., 2015). This wide range likely reflects the large number of questions that have been used to define tinnitus, which makes the genetic basis of tinnitus difficult to determine. An age-dependent increase in the prevalence of tinnitus is seen across all studies, with a peak in the seventh decade of life (Gopinath et al., 2010; Shargorodsky et al., 2010; Park et al., 2014). There is no agreement on whether there is a gender bias, but there is a tendency for males to be more affected than women (Cooper, 1994; Sindhusake et al., 2003; Hasson et al., 2010; Krog et al., 2010; Michikawa et al., 2010; Nondahl et al., 2010; Shargorodsky et al., 2010; Engdahl et al., 2012; McCormack et al., 2014; Park et al., 2014; Gallus et al., 2015).

With regard to ethnic differences, studies performed in Egypt (Khedr et al., 2010), Japan (Michikawa et al., 2010), and Nigeria (Lasisi et al., 2010) suggest that the prevalence is broadly the same. However, one study reported a higher prevalence of tinnitus in non-Hispanic whites than in other racial or ethnic groups in the U.S. (Shargorodsky et al., 2010). Additional ethnic studies are needed to infer potential genetic influences on tinnitus.

Table 1 presents a summary of human genetic studies for tinnitus. Most of them were genotyping studies with a small sample size (54–288) on candidate genes including *KCNE1, KCNE3, GDNF, BDNF, COCH*, and *SLC12A* (Sand et al., 2010, 2011, 2012a,b; Gallant et al., 2013). Overall, no associations were found with one exception (Pawelczyk et al., 2012). The small sample size and the paucity of patient characterization (tinnitus only being characterized as chronic) could account for these outcomes. For instance, the study by Sand et al. (2010) included 201 German patients with “chronic tinnitus” and no controls. The authors used public genotyping data from other studies as control subjects, without any ancestry-informative markers to prevent population stratification. Pawelczyk et al. (2012) conducted a case-control study in Poland including 626 subjects exposed to occupational noise (128 with tinnitus and 498 without tinnitus). While they reported an association with the SNP rs915539 in normal hearing subjects (*p* = 0.005), no ancestry-informative markers were used and the current standards in genetic association studies require a replication in another association study with an independent population, something that to our knowledge has not been yet reported.

**Table 1:** **Available human genetic studies on tinnitus**.

**Tinnitus property**	**HL**	**Size of the population**	**Reported gene**	**Design**	**Associations**	**References**
Tinnitus associated with NIHL	Occupational Noise	*N* = 626 (128 with tinnitus)	KCNE1, SLC12A2	Genotyping	KCNE1 associated with tinnitus independent of HL	Pawelczyk et al., 2012
Chronic	Controlled	*N* = 240	GDNF, BDNF	Genotyping	None	Sand et al., 2012b
Chronic	Controlled	*N* = 95	KCTD12	Genotyping	None	Sand et al., 2012a
Chronic	Not reported	*N* = 201	KCNE1	Genotyping	None	Sand et al., 2010
Unknown	All	*N* = 54	SLC6A4	Genotyping	None	Deniz et al., 2010
Chronic	Not reported	*N* = 288	KCNE3	Sanger sequencing	None	Sand et al., 2011
Tinnitus associated with HFHL	HFHL	*N* = 1 family	COCH	Linkage analysis	Single family study	Gallant et al., 2013
Unknown	All	*N* = 28,066	None	Familial aggregation	Population-based study	Kvestad et al., 2010
Unknown	Not reported	*N* = 198 families	None	Familial aggregation	Multiple family study	Hendrickx et al., 2007

*HL, Hearing loss*.

Studies on familial tinnitus are scarce. A large study analyzed the occurrence of familial tinnitus within 198 European families (Hendrickx et al., 2007). The authors found a familial correlation between siblings reaching 0.16, and the finding was independent of differences in age, gender, and hearing threshold. Using a Cox proportional model, the risk of developing tinnitus was estimated to be 1.7 times higher in siblings with tinnitus than that observed in families without tinnitus, after correcting for risk factors (Hendrickx et al., 2007). However, the authors reasoned that this could be simply due to the fact of raising awareness on tinnitus within the family. The selection of multicase families with tinnitus for exome sequencing studies to search for rare variants with a high penetrant effect has not been explored.

To the best of our knowledge, there is no published work on the concordance, or heritability of tinnitus from twin studies. Such studies could appropriately address the issue of sibling influences on awareness and provide solid evidence on whether or not there is a genetic contribution to tinnitus. Heritability is an estimation of the genetic contribution in relation to the phenotypic variability for a particular trait that occurs within populations. The variation in the phenotype for a particular trait in a population arises from differences in the genotype and environmental variation. Falconer's formula for estimating heritability is based on the concordance rates among monozygotic and dizygotic twins:

(1) $$\begin{array}{l} {\text{h}}^{2}=2^{*}\left(\text{rMZ}-\text{rDZ}\right) \end{array}$$

where h^2^ is the heritability or the proportion of variance due to genetic factors and r is the correlation coefficient between MZ and DZ twins. Heritability values have a theoretical range of 0–1.5. In general, it is considered that a trait has a genetic component if h^2^ is between 0.5 and 1. With this approach in mind, we have initiated a study to evaluate the concordance of tinnitus in twins and ongoing data collection is in support of a genetic contribution to some forms of tinnitus.

## Tinnitus phenotyping: needle in a haystack?

A major limitation in genetic association studies, whatever the field of research, is the classification of subjects according to a common phenotype. Tinnitus is considered a symptom. It is thought that the large number of clinical conditions associated with chronic tinnitus has contributed to the unsuccessful clinical trials and genetic studies listed above. An initial suggestion of classification into subgroups was proposed by the Tinnitus Research Initiative in 2010 (Landgrebe et al., 2010) followed by the Tinnitus Holistic Simplified Classification (Cianfrone et al., 2015). The Tinnitus Holistic Simplified Classification proposes that tinnitus stems from (i) auditory alterations (Auditory Tinnitus), (ii) complex auditory-somatosensory interactions (Somatosensory Tinnitus), (iii) psychopathological-auditory interactions (Psychopathology-related Tinnitus), and (iv) 2 or all of the previous mechanisms (Combined Tinnitus). Others have classified tinnitus into originating either from the auditory system (usually peripheral, rarely central) or from the somatosensory system (head and neck), or a combination of the two (Levine and Oron, 2015). Recently, another work has revealed that somatic tinnitus may represent a subtype (Ward et al., 2015), being more prevalent in younger groups, unrelated to hearing loss but rather associated with TMJ disorders. Overall, the definition of tinnitus subtypes is still a matter of debate, and no consensus has been found due to the large number of contributing factors, the multitude of etiologies, and the psychoacoustic profiles of tinnitus.

The benefits of subtyping approaches in genetic studies have been shown in a genome-wide association study (GWAS) for major depressive disorders (MDD). The analysis of more than 9000 cases did not yield robustly replicated genetic loci, and it was thought that the heterogeneity contributed to the reduction in the power of the genetic associations. The selection of a severe subtype of MDD with accompanying melancholia allowed the successful mapping of a single gene, namely *SIRT1* (CONVERGE, 2015). Such stratification of diseases into homogeneous subcategories or subtypes has been successful in reducing genetic background noise and clinical heterogeneity, ultimately helping in the identification of genetic variants (Gelernter et al., 2006; Schwartz et al., 2010). Although these approaches may lead to hits that are not applicable to the general population, they may still facilitate (i) the understanding of the mechanisms of specific subcategories of tinnitus, (ii) the development of biological markers of tinnitus subtypes, and (iii) the identification of candidates for drug development.

How can the tinnitus field benefit from genetic studies to improve treatment outcome? While such conceptual approaches are at the forefront of disease treatment, an example can be provided with ongoing research on a specific subtype of Autism Spectrum Disorder (ASD), namely Phelan-McDermid syndrome, which is a rare disorder with deletions or mutations in the SHANK3 gene. Studies have shown the beneficial use of IGF-1 for neuronal function using cells with the SHANK3 mutations (Bozdagi et al., 2013). This approach has been tested on nine children in a pilot study showing the successful therapeutic effects of IGF-1 treatment in improving social behavior and reducing repetitive behavior (Kolevzon et al., 2014), whose positive outcomes have also been reported in a preclinical mouse model of autism (Bozdagi et al., 2013). Such studies show how genetic studies, coupled with preclinical research, can help in developing targeted treatments for different disease subtypes. Such examples are on rare monogenic disorders, so how can this be applied to tinnitus, which—assuming there is significant heritability—would likely be polygenic? Tinnitus is probably a polygenic condition, however genomic research will reveal whether some subtypes of familial tinnitus, are monogenetically driven.

Several disorders have been categorized into subgroups in order to facilitate the identification of biomarkers and optimize treatment outcomes. Schizophrenia is segregated into subtypes according to the expression of behavioral symptoms (e.g., paranoid, disorganized, catatonic, undifferentiated, and residual). Multiple sclerosis (MS) subtypes, on the other hand, are defined on the basis of time-course development (primary progressive, relapsing-progressive, relapsing-remitting, secondary progressive, transitional progressive), prognosis, and pathogenicity (obtained through the analysis of biopsies) (Bitsch and Brück, 2002). Interestingly, in the case of MS, studies have revealed blood, CSF, and MRI biomarkers associated with particular subgroups. Alzheimers disease is categorized into three subgroups thanks to metabolic profiling (Bredesen, 2015): inflammatory (presence of specific blood markers), noninflammatory (absence of these blood markers), and cortical (no specific Alzheimer gene detected, but normally associated with zinc deficiency). The value of these biomarkers in clinical practice remains to be established due to the large phenotypic variability.

The above examples possess numerous advantages over tinnitus. First, these are diseases whereas tinnitus is considered a symptom. Second, they rely on available biomarkers from blood, CSF, molecular, and histological profiles. An example of the advantage that these biomarkers provide to the refinement of genetic studies has been shown in bipolar disorders. Bipolar disorders are classified into two major subtypes. Kynurenic acid (KYNA) has been recently identified as a CSF biomarker in both subtypes, and has been associated with a greater history of psychosis. This biomarker provided a powerful advantage in a recent GWAS study involving only 76 patients and 46 controls that identified a single nucleotide polymorphism causing a reduction of sorting nexin 7 (*SNX7*) expression in astrocytes, leading to higher IL-1β production, and subsequently increasing KYNA in patients carrying this variant (Sellgren et al., 2015).

The tinnitus field suffers from a lack of such biomarkers. Current subtyping strategies thus rely on the clinical features of tinnitus (acute vs. chronic, objective vs. subjective, pulsatile vs. nonpulsatile, constant vs. intermittent), taking into account cofactors such as hearing loss, vertigo, headache, psychiatric influences, and somatosensory origins, as well as its triggers (e.g., noise trauma, accident, medication, Ménière's disease). A putative list of factors that need to be taken into account is shown in Table 2. Which of these are relevant to tinnitus will only emerge in future clinical and genetic studies.

**Table 2:** **List of potential factors to take into account in genetic studies on tinnitus**.

**Forms of tinnitus**
Subjective, objective
Pulsatile, nonpulsatile
Constant, intermittent
Unilateral, bilateral
**Temporal**
Acute, subacute, chronic
**Severity**
Moderate, severe, catastrophic
**Etiology**
Noise trauma, medication, post-traumatic stress disorder, Ménière's disease, TMJ
**Influencers**
Age, sex, ethnicity
**Cofactors**
Hearing loss, hyperacusis, vertigo, headache, psychiatric (stress, anxiety, depression), somatosensory
**Comorbidities**
Hypertension, diabetes, cancer, chronic pain, neurological problems,
**Response to treatment**
Improvement, worsening, none

*We propose a nonexhaustive list of factors to take into account when designing genetic studies on tinnitus. A large variety of tinnitus subtypes may thus emerge from the combination of severity, forms of tinnitus, etiology, temporal characteristics, and comorbidities*.

The selection of individuals for genetic studies will have to consider all the above features, including severity, duration, gender, age, age of onset, pitch, intensity, hearing thresholds, psychological burden, and etiology, to reduce clinical heterogeneity, and to control biases. Categorizing tinnitus subtypes according to tinnitus pitch, severity, and hearing profile might be sufficient, however this needs to be tested. In addition, tinnitus perception may change over time and patients might be classified into a different subtype, or even belong to multiple subtypes (e.g., noise trauma causing unilateral deafness, being initially acute, and then transiting to chronic stages, becoming bilateral with the emergence of psychiatric burden but still unilaterally dominant, and pitch decreasing with age). As a consequence, psychoacoustic evaluations should be performed in the first years of the onset of tinnitus to reduce the number of confounding factors. Finally, there is little biological information on the mechanisms underlying each of these subtypes, and this is where genetics may play an important role by defining a subgroup of tinnitus subjects with a defined phenotype. The identification of tinnitus subtypes is thus in the early stages.

## Pathophysiology of tinnitus

### Mechanisms mediating tinnitus perception

**Figure 1:**
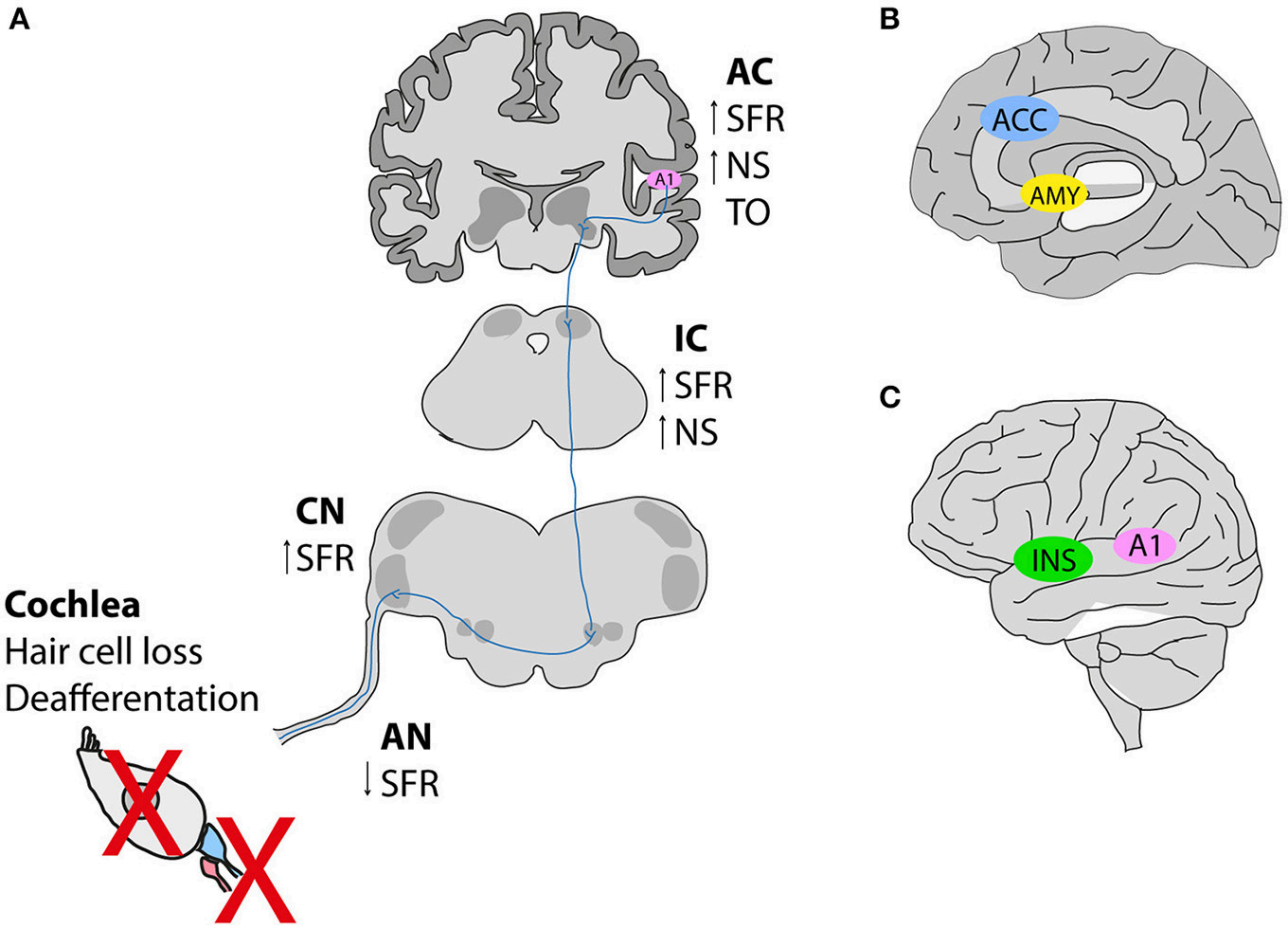
**Hypothetical schema of tinnitus pathogenesis. (A)** Noise exposure in animal models leads to deafferentation between inner hair cells and afferent neurons, or hair cell loss subsequently causing a reduction in the spontaneous activity of the auditory nerve (AN). In contrast, an increase in the spontaneous firing rates is observed along the auditory pathway [dorsal cochlear nucleus (CN), inferior colliculus (IC) and auditory cortex (A1)]. Tonotopic organization (TO) is also altered in the auditory cortex **(B,C)**. Neuroimaging studies in humans with tinnitus identified the involvement of nonauditory areas such as the salience network [the anterior cingulate cortex (ACC) and the insula (INS)], as well as the emotional components of tinnitus involving an increased connectivity between the amygdala (AMY), and the primary auditory cortex (A1). SFR, spontaneous firing rate; NS, neuronal synchrony. This figure was modified from Elgoyhen et al. (2015) with permission from the Nature Publishing Group (Elgoyhen et al., 2015).

A detailed phenotyping of tinnitus patients is necessary to investigate the genetic and environmental factors contributing to its development. Both auditory and psychological components of tinnitus are important aspects to be evaluated. Studies reveal that tinnitus possesses a dual mechanism that emerges most frequently from (i) peripheral dysfunctions leading to changes in (ii) the activity of the central auditory pathway likely influenced by nonauditory networks that feed tinnitus-related distress and possibly influence its persistence (Figure 1). The current knowledge stipulates that the perception of tinnitus resembles the phantom perception of an amputated limb, whereby the loss of sensory input leads to compensation mechanisms in the brain (hyperactivity). Indeed, tinnitus networks are similar to those involved in chronic pain (perception, salience, distress, memory), and could contribute to the maintenance of tinnitus, in the absence of the initial trigger (Langguth et al., 2013). Confirming the idea about the loss of peripheral (cochlear) input causing tinnitus, human subjects that wore a silicone earplug for 7 days experienced tinnitus (Schaette et al., 2012), which disappeared after the earplug was removed, supporting the hypothesis that therapeutic interventions restoring cochlear output to the brain can abolish phantom perception.

The relationship between peripheral damage and tinnitus has been recently reviewed (Schaette, 2014). Patients with conductive hearing loss (e.g., otosclerosis) often complain about tinnitus, which is then completely abolished after surgery (Gersdorff et al., 2000; Ayache et al., 2003; Sobrinho et al., 2004). Similarly, hearing aids, and cochlear implants are capable of improving tinnitus in 50% of patients, and abolishing tinnitus in 20% of cases (Moffat et al., 2009; Olze et al., 2012; Schaette, 2014). Mertens et al. provided the only long-term study that clearly shows a reduction in tinnitus and hypercusis with cochlear implants (Mertens et al., 2016). Interestingly, one study reported lower amplitudes of wave I recorded from click auditory brainstem responses (ABRs) in tinnitus patients with normal hearing thresholds when measured by pure tone audiometry, suggesting the existence of cochlear damage leading to a decreased input toward the brain (Schaette and McAlpine, 2011). Overall, these studies suggest a peripheral (cochlear) contribution in some forms of tinnitus, which supports the inclusion of ABR measurements in patients with normal audiometry. Central mechanisms that compensate for the lack of input (homeoplastic plasticity) could emerge. Interestingly, fMRI studies revealed that people with tinnitus have increased activity in auditory, and nonauditory networks such as the limbic system, including the nucleus accumbens (Rauschecker et al., 2010; Leaver et al., 2011). It was suggested that this increased activity was a result of reduced functional output of the ventromedial prefrontal cortex in tinnitus patients (Leaver et al., 2011). Activation of the nucleus accumbens would lead to increased inhibition of thalamic reticular nucleus neurons, and thus result in increased inhibition of medial geniculate body neurons. In patients with gaze-induced tinnitus, hypometabolic theta activity, and reduced inhibition in the auditory cortex were found to occur hand in hand with reduced medial geniculate body activity (van Gendt et al., 2012). However, the precision of EEG measures in tinnitus assessment has recently been questioned (Pierzycki et al., 2016). It can be concluded that there is clear evidence of a profound impact of the “emotional brain network” on the generation of manifestation of tinnitus.

### Mechanisms mediating tinnitus-related distress

Most people have probably transiently experienced tinnitus at some point in their life. However, in some cases tinnitus becomes permanent and can seriously impact the quality of life. Interestingly, in individuals with chronic persistent, nonfluctuating tinnitus, the psychoacoustic characteristics of tinnitus (e.g., loudness or pitch) are not unequivocally related to its severity or the treatment outcome (Jastreboff and Hazell, 1993). In chronic tinnitus, *the interpretation of the tinnitus percept* might be more important in impacting the severity of complaints than the sound itself (Jastreboff and Hazell, 1993; Henry and Meikle, 2000; Andersson, 2003; Hiller and Goebel, 2007). Psychological distress, which includes negative attitudes, and cognitions, impaired concentration, insomnia, depression, and anxiety, is a significant predictor for the variability in the quality of life (Erlandsson and Hallberg, 2000). Accumulating evidence suggests that cognitive misinterpretations, negative emotional reactivity and attention processes are crucial in dysfunctional habituation leading to severe tinnitus distress (Erlandsson and Hallberg, 2000; Kröner-Herwig et al., 2003; Zachriat and Kröner-Herwig, 2004; Cima et al., 2012).

The emotional neural networks that possibly influence the peripheral to central circuit in tinnitus patients likely comprise the regions known to be involved in normal emotional behavior. These regions can be altered in mood disorders and involve the medial prefrontal cortex, the medial, and caudolateral orbital cortex (medial prefrontal network), anterior cingulate, amygdala, hippocampus, and ventromedial parts of the basal ganglia (Jastreboff, 1990; Drevets et al., 2008). Indeed, clinical imaging of individuals with tinnitus provides evidence that tinnitus-related and distress-related brain networks overlap, such as the limbic, and paralimbic regions (Rauschecker et al., 2010), the amygdala (Shulman, 1995; Mirz et al., 2000), the hippocampus (Lockwood et al., 1998; Landgrebe et al., 2009), the basal ganglia (Lowry et al., 2004; Cheung and Larson, 2010) and the subcallosal region, including the nucleus accumbens (Mühlau et al., 2006; Leaver et al., 2011). Favoring the possible cross-modal interactions of the limbic system central responsiveness, perhaps related to the peripheral damage after auditory trauma, thalamic/amygdala projections change their activity pattern during tinnitus (Knipper et al., 2013). Overall, it appears to be important to measure emotional components during tinnitus phenotyping.

## Tinnitus phenotyping strategies

Precise phenotyping of patients with tinnitus is the first step in defining clusters of patients based on a few variables that will configure a tinnitus subtype (Tyler et al., 2008). Poor phenotyping can significantly contaminate large epidemiological or genetic studies leading to a loss of power and false-positive results. For instance, not controlling for emotional factors (such as stress, anxiety, or depression) could lead to the identification of genes falsely associated with tinnitus, while they would be truly linked to depression. Hearing profile and tinnitus pitch are minimum requirements, but additional measures—including questionnaires covering psychological aspects—are also needed. The common psychological comorbidities of depression, anxiety, insomnia and cognitive impairment disable 10–50% of patients suffering from tinnitus. Similarly to some tinnitus measures, assessment of tinnitus comorbidities has been neglected in drug development efforts. This gap is currently being addressed in a consensus-driven effort to provide international guidelines on Core Outcome Measures in Tinnitus (COMiT) (Hall et al., 2015), which will define the domains and related instruments necessary to perform tinnitus studies.

An example that illustrates the importance of genetic studies in subtypes of tinnitus patients is the identification of a polymorphism in the serotonin transporter gene (*SLC6A4*), which has been previously shown to be associated with anxiety (Lesch et al., 1996), and is now linked with the severity of the psychological conditions associated with tinnitus (Deniz et al., 2010). As a consequence of these findings, one could envisage that *SLC6A4* variants could become markers of tinnitus distress, and that serotonin reuptake inhibitors could be targeted at subtypes of patients with tinnitus and depression in the presence of the risk allele. However, some of the mechanisms and drug treatments of these tinnitus comorbidities might differ from patients without tinnitus, which would suggest tinnitus-specific mechanisms.

Defining potential tinnitus subtypes will be essential in investigating the heritability for each subtype in familial and twin studies. This strategy will enhance the results in genetic studies, in addition to improving clinical trial outcomes. However, this can be a challenging task since a subtype will also be characterized by either a successful therapeutic intervention or by the identification of a gene associated with, for example, a particular form of tinnitus. In the context of genetics, this conundrum can be potentially addressed with concordance studies in twins by identifying traits that are more prevalent in monozygotic twins than in dizygotic twins.

The assessment of a patient with tinnitus should include a complete audiological evaluation, psychoacoustic measures of tinnitus and several instruments to determine the severity of tinnitus, and its impact on health-related quality of life. However, it is important to note that the exclusion of measures could also lead to the inclusion of nonspecific groups and bias the genetic analysis or treatment outcome. A comprehensive measure of tinnitus features is thus required to characterize each form of tinnitus. To achieve this important classification procedure, considerable thought should be invested in selecting the right tools for measuring tinnitus experience (e.g., validated questionnaires, psychoacoustic measures, audiological measures), and the selection will depend on the aims of the study.

A number of instruments have been recommended by the Tinnitus Research Initiative (Langguth et al., 2007) for the assessment of treatment outcomes in clinical trials. Of note, we emphasize that conventional pure tone audiometry (PTA), which measures hearing thresholds from 250 Hz to 8 kHz, is no longer adapted to tinnitus cases. A number of tinnitus patients diagnosed with no hearing loss when measured with conventional audiometry tend to be diagnosed with somatosensory tinnitus. However, high-frequency PTA (up to 20 kHz) might reveal an auditory component to tinnitus, thereby completely reallocating a patient into another subtype category. Normal hearing should be considered from <20 dB HL up to 16 kHz in adults. Interestingly, one study reported lower amplitudes of wave I (based on I/V ratio) recorded from click auditory brainstem responses (ABRs) in tinnitus patients with normal hearing thresholds, however the latter were measured with PTA only up to 12 kHz, suggesting that a decreased cochlear input toward the brain causes some forms of tinnitus (Schaette and McAlpine, 2011). ABRs could thus become important in revealing cochlear damage and objectively categorize subjects into a specific peripherally injured tinnitus subtype. However, since ABRs are known to be sensitive at higher frequencies (Don and Eggermont, 1978; Eggermont and Don, 1980), differences in hearing thresholds above 12 kHz could have accounted for these wave I/V differences in amplitude in the tinnitus group. This reinforces the importance of assessing PTA up to at least 16 kHz. Distortion products of otoacoustic emissions (DPOAEs) are measures of outer hair cell function. Often neglected in the assessment of tinnitus patients, DPOAEs can measure both a decreased function and a loss of outer hair cells (OHCs) likely due to cell death, or a gain in OHC function as can sometimes be observed in subgroups of tinnitus patients with hyperacusis (Sztuka et al., 2010). Psychoacoustic measures have been most commonly used to determine the pitch-matched frequency and intensity of the perceived tinnitus. Little is known on how tinnitus pitch can evolve with time and whether patients with different pitches might constitute different subtypes. Neuroimaging studies have been recently reviewed (Elgoyhen et al., 2015) and it has been proposed that tinnitus heterogeneity is the consequence of abnormal activity from specific networks. Neuroimaging techniques, including fMRI, EEG, and MEG, could thus constitute an important set of instruments to help categorize tinnitus patients into different subgroups according to the involvement of specific networks (such as the hippocampal-cortical memory networks, the frontoparietal control system, the salience network, and the autonomic nervous system). Research is currently underway to define which networks specify a given subtype of tinnitus and how relevant these tools can be for characterizing tinnitus.

## Designing human genetic studies

Over the last three decades, medical genetic research has focused largely on inherited variation in the human genome. Most of the DNA variability can be explained by single nucleotide variants (SNVs) and small structural variants involving one or a few nucleotides (insertions, deletions), or large structural variants involving hundreds to thousands of nucleotides (copy number variants, CNVs). These variants mostly occur in noncoding regions, which can affect the degree of expression of a given allele, but CNVs may also involve coding regions causing partial or complete loss or gain of function.

There are several complementary approaches to demonstrate an association between genetic variants and tinnitus in humans:

- focusing on patients with a common genetic background (e.g., identical twins or familial aggregation studies) to estimate heritability.
- designing case-control studies (e.g., cases with common etiology or disease such as Ménière's disease) to search for rare variants on monogenic tinnitus families.
- GWAS using large cohorts of sporadic patients to search for common regulatory variants.

All of these designs can be used to identify the most heritable tinnitus phenotype and to find candidate genes. However, the complexity and heterogeneity of tinnitus implies the need for in-depth tinnitus phenotyping, using questionnaires as well as audiological and psychoacoustic measures, to accurately identify genes responsible for tinnitus resilience, or susceptibility.

### Methods: genotyping vs. sequencing

There are two methods for reading the genome: genotyping and sequencing. Genotyping determines the differences in SNVs in a given individual when their sequence is compared with the reference genome. Sequencing is the process of determining the nucleotide order of a given DNA fragment and is usually performed for short fragments of DNA by the chain termination method developed by Sanger et al. (1977). New sequencing technologies such as pyrosequencing have enabled rapid, large-scale sequencing of human genome, including whole genome sequencing (WGS) and the most popular enrichment approach for coding regions, whole exome sequencing (WES) (Mardis, 2008).

Genotyping larger cohorts of patients with a given disorder using microarrays has been the basis for GWAS during the last 15 years. Trait-associated SNVs have identified regulatory common genetic variants (minor allele frequency—MAF > 0.05) with small genetic effects, but are unlikely to define the causative rare variants in most cases. Although GWAS for complex disorders have resulted in great progress, most of the candidate genes investigated in case-control studies, including candidate genes for chronic tinnitus, could not be replicated. Replication is essential for establishing the credibility of a genotype—phenotype association, whether derived from candidate genes or GWAS (Mardis, 2008). Large-scale genotyping studies are based on the knowledge that SNVs along the entire genome are conserved in specific regions, and SNVs can be used as markers of the sequence in these regions. To generate a map of SNVs in the human genome, the HapMap Project was carried out (International HapMap, 2003). GWAS have identified common SNVs in large-population studies, mostly in noncoding regions with unknown functional significance (Cooper and Shendure, 2011). Furthermore, this design is not suitable for the study of genetic conditions that are caused by rare or novel mutations (Robinson et al., 2011).

On the other hand, high-throughput sequencing technologies, such as WES, are designed to enrich the sequencing of coding regions, which contain 85% of disease-causing mutations defining rare variants in familial and sporadic patients in 65% of cases (Samuels et al., 2013). Moreover, the cost of WGS or WES studies has been dramatically reduced in recent years, facilitating their implementation for clinical diagnosis (Biesecker and Green, 2014). Although genotyping has been the preferred approach to identify common SNVs with regulatory effects in GWAS, the decreased cost involved in WGS is predicted to lead to genotyping being replaced in a few years. WES and WGS have become the standard in searching for rare variants in any genomic study.

### Candidate gene vs. genomic approaches

Several genes have been considered as candidate genes for tinnitus, but replication studies, are missing or have failed to confirm previously reported associations (Table 1). The main reason for the lack of reproducibility is population stratification or the systematic ancestry differences between cases and controls, which is a confounder in genetic association studies (Price et al., 2010). Instead, targeted sequencing of candidate genes is considered a suitable method to determine the relevance of a candidate variant previously identified by a genomic approach.

Genotyping microarrays and next-generation sequencing technologies help to overcome the limitations of traditional approaches. Either WGS or WES combined with linkage studies have become the most efficient strategies for discovering causal genes for Mendelian diseases (Zhang, 2014). We have used this approach to identify novel and rare variants in *FAM136A* and *DTNA* genes in autosomal dominant familial Ménière's disease (Requena et al., 2015). We were also able to reveal a missense variant in the *PRKCB* gene in a family with Ménière's disease segregating low-frequency sensorineural hearing loss (Martín-Sierra et al., 2016). The next step will be to investigate rare variants of candidate genes in more families and sporadic cases. This approach can be used for specific forms of familial tinnitus after obtaining a detailed phenotype. To the best of our knowledge, this strategy has not been applied yet to specific tinnitus subtypes. The clinical heterogeneity of tinnitus makes the selection of patients according to the tinnitus phenotype a crucial step in the design of the study.

### Sample selection

There are compelling reasons to focus on tinnitus symptoms that are defined by a common trigger or clinical syndrome. First, the more homogenous the tinnitus phenotype, according to the tinnitus pitch and hearing profile, the better the chance that an allelic variant segregates with the particular phenotype. The reason to classify tinnitus by its frequency is the tonotopic gradient of gene expression in the mammalian cochlea (Yoshimura et al., 2014). The frequency selectivity is maintained along the auditory pathway and precise regulation of this gene expression is required to preserve tonotopy. Individuals with selective low- or high-frequency sensorineural hearing loss could potentially be good candidates for a case-control study. The reduction in error will increase the power to detect a small gene effect. Second, patients with different tinnitus conditions will vary in other ways that increase the variance and reduce the power to detect gene effects. For instance, a completely different set of factors may mediate the onset of chronic tinnitus due to age-related hearing loss vs. an ear injury or a cardiovascular disorder. For this reason, the selection of younger individuals is preferred, since the cumulative effect of different epigenetic and environmental triggers may favor the onset of tinnitus in elderly individuals. Third, completely different measures are needed to adequately characterize a tinnitus phenotype in different conditions, for example, in noise-induced tinnitus vs. stress-induced tinnitus.

There are several limitations when designing genetic studies in patients with chronic tinnitus. First, the clinical heterogeneity observed makes it difficult to select patients with the same phenotype (Sand et al., 2007). A clinically well-defined phenotype is a prerequisite in designing a case-control study. Since most patients with tinnitus also have a certain degree of hearing loss and a number of comorbidities related to tinnitus, the design should control these biases by selecting individuals with the same hearing profile and tinnitus pitch. Since it has been hypothesized that tinnitus subjects will possibly accumulate multiple common and rare variants segregating with the phenotype, it could be advantageous to select younger individuals in multicase families in order to search for highly penetrant rare variants with a large effect size (Requena et al., 2014). In contrast, older subjects with tinnitus will probably reflect the cumulative effect of epigenetic and environmental factors throughout their lives, diluting the effect of genetic variation. Therefore, a possible solution is to reduce the selection to a subset of patients with extreme phenotypes, filtering them according to early age of onset, gender, ethnic background, and for instance, specific clinical features that would show higher concordance in monozygotic twins. Such strategies have proven successful in previous studies, whereby the exclusion of hearing impairment increases the number of twins concordant for noise sensitivity (Heinonen-Guzejev et al., 2005) and the selection of a subtype of severe melancholia increases the concordance of major depressive disorder (CONVERGE, 2015). Furthermore, the selection of a reference population matching for age, gender, ethnicity, comorbidities, emotional burden, and quality of life could also be critical, since many confounder factors may arise.

A second limitation for small-size case-control studies is that tinnitus is a highly prevalent condition, which anticipates that many genetic variants could confer resilience or susceptibility (Veltman and Brunner, 2012). A large genetic heterogeneity is expected for chronic tinnitus, which would possibly complicate the functional interpretation of rare variants in genes encoding, for instance, proteins that are known to have a physiological role in the synapse. Often associated with tinnitus is the high-frequency SNHL that is typically observed in presbycusis and is known to have a significant genetic heterogeneity (Fransen et al., 2015).

Learning from previous research in fields such as pain and schizophrenia, which are very heterogeneous disorders, we believe that studies should be restricted to the most homogeneous groups in terms of etiology, age, gender balance, severity of tinnitus, audiometric profile, and comorbidities. Moreover, the smaller the variation of the genetic background within a group, the more robust the study will be. In this direction, we favor the following sequence of prioritization: studying twins > multiplex families with tinnitus > Ménière's disease patients with chronic tinnitus, groups with cisplatin-induced tinnitus following chemotherapy, noise overexposure (military training or work exposure), or ARHL (age-related hearing loss) > large health cohorts with undefined etiology.

### Design of tinnitus sequencing studies

Let's consider one example for which the initiating trigger is well-defined (i.e., noise trauma or Ménière's disease), and another example where the initiating trigger is not clearly defined. After sensorineural hearing loss, some patients experience short-term tinnitus, but do not develop chronic tinnitus, suggesting some type of resilience. However, a few patients with some intrinsic susceptibility will experience chronic persistent tinnitus.

In a case-control design, the case group is defined as having chronic persistent tinnitus as a consequence of the trigger, and controls must have had the initiating trigger as well, leading to temporary tinnitus, or no tinnitus. Data collected from cases and controls can include previous tinnitus history, history of psychiatric disorders, assessment of traits, exposure to stressors, and actual comorbid conditions relevant to tinnitus domains (hearing loss, hyperacusis, stress, anxiety, or depression). Then, cases and controls are compared at the level of individual domain-specific measures. Measures from different domains can also be compared (e.g., hearing loss and stress) in order to better understand the subgroups. Such comprehensive studies will accelerate the gathered knowledge on the interaction between causative factors and the genetic components underlying a specific tinnitus phenotype.

Obviously, the method chosen for the case-control studies will depend on the incidence, prevalence of the condition and the proportion of those with the phenotype of interest that seek care. Direct ascertainment in the population might differ from the phenotypes assessed in tinnitus clinics (self-selected samples) since the psychiatric conditions and behaviors of those who seek care can be genetically influenced. As an example, treatment resistance, and psychiatric comorbidities are more likely to occur in patients with migraines that get treated by a specialist than in a population-based sample for the same disorder (Lipton et al., 2003; Kolodner et al., 2004; Bigal et al., 2006). In addition, identifying appropriate controls for the tinnitus groups from specialty care centers can be particularly challenging.

### Epigenetic factors possibly contributing to tinnitus

Epigenetics is the discipline that studies changes to the genome that do not involve modifications in the DNA sequence *per se* (Cederroth et al., 2007). Since psychological distress is often associated with tinnitus, and psychosocial stress has been well documented in animals and people as a modifier of epigenetic marks (Franklin et al., 2012; Bohacek and Mansuy, 2015; Vaiserman, 2015), it is tempting to speculate that tinnitus could also emerge from epigenetic modifications. The two main epigenetic mechanisms are gene methylation and histone modifications. DNA methylation typically reduces or even silences the expression of genes encoded by methylated DNA. The modification of histones may either enhance or reduce gene expression, depending on the type of histone and type of modification. Histones, which are structural proteins of chromatin, are responsible for tight packaging of DNA, and their modifications (e.g., acetylation or deacetylation) affect the accessibility of DNA by various enzymes. Changes in methylation occur during embryonic development as early as a few hours in the paternal genome after fertilization, whereas in the maternal genome this is a more passive phenomenon. After the implantation of the embryo, along the differentiation of embryonic tissues, cells become more abundantly methylated—a phenomenon called reprogramming (Jaenisch, 1997; Mayer et al., 2000). In adulthood, the environment can induce changes in specific cell types. Monozygotic twins offer an excellent illustration of this phenomenon, since despite their genetic identity, there are morphological variations and also different susceptibility to diseases. Environmental factors such as psychosocial stress, smoking, physical activity, or diet can contribute to such epigenetic drifts.

In animal models, restraint stress, acute forced swim stress, social isolation stress, and many other types of stress can induce epigenetic modifications, such as on the loci of the glucocorticosteroid receptor (GR) or brain-derived neurotropic factor (BDNF) (Fuchikami et al., 2010; Stankiewicz et al., 2013). There are many studies showing that experimentally induced behavioral changes are linked to these epigenetic modifications; this was also observed in people suffering from depression or anxiety (Bagot et al., 2014).

To date, no published studies have focused on possible epigenetic aspects of tinnitus onset or progression. However, some studies have indirectly approached this topic in the context of hearing loss (Provenzano and Domann, 2007; Wolber et al., 2014). For instance, the pattern of gene methylation in a group of patients with age-related hearing impairment was found to differ from that found in well-hearing subjects (Wolber et al., 2014). Because the incidence of hearing loss in tinnitus patients is high, it would be tempting to speculate that at least some of the epigenetic targets may overlap between the two conditions (Goldman and Holme, 2010; Mazurek et al., 2010). In addition, the comorbidity of psychological conditions such as anxiety or stress (Hébert and Lupien, 2007; Hébert et al., 2012) may possibly create a disease-specific pattern of epigenetic modifications.

The epigenetic modifications often affect specific tissues, but not the entire organism, which renders the study of human auditory tissues challenging (e.g., inner ear and central auditory pathway) due to their limited access. However, epigenetic modifications could occur during fetal development (e.g., maternal stress during gestation)—a phenomenon called “fetal reprogramming” (Moisiadis and Matthews, 2014). Then, peripheral tissues might be used as a proxy for brain-specific alterations (Stenz et al., 2015). Finally, if they occur during adulthood, the consequences of the insults can also be found across generations both at the level of the phenotype and in the male germline epigenome (Anway et al., 2005; Franklin et al., 2010). These possibilities offer new routes for investigating the relationship between tinnitus and epigenetic changes related to comorbid conditions such as stress, anxiety, and depression.

## Conclusions

Human genetic studies in tinnitus are at the very beginning. Accordingly, concordance studies in twins are an essential first step in defining the heritability of tinnitus. In a second step, the precise selection of subjects based on careful phenotyping will facilitate the identification of genes involved in the resilience or susceptibility to developing tinnitus or tinnitus-related comorbidities. The molecular characterization of tinnitus will not only lead to a better understanding of the pathways and networks regulating the onset of disease, but also shed light on the physiological processes involved, leading to the development of new pharmacological treatments.

## Author contributions

JL, CC, TB, RC, PV, MK, BM, AS, contributed to the manuscript. CC, JL, and CC coordinated the writing, designed the tables and figures, and edited the manuscript.

### Conflict of interest statement

The authors declare that the research was conducted in the absence of any commercial or financial relationships that could be construed as a potential conflict of interest. The Handling Editor is collaborating with some of the authors of the manuscript as part of a Research Topic and the Handling Editor states that the process nevertheless met the standards of a fair and objective review.
